# Women's preconception psychological stress and birth outcomes in a fertility clinic: the EARTH study

**DOI:** 10.3389/fgwh.2024.1293255

**Published:** 2024-02-05

**Authors:** Lidia Mínguez-Alarcón, Paige L. Williams, Irene Souter, Jennifer B. Ford, Russ Hauser, Jorge E. Chavarro

**Affiliations:** ^1^ Channing Division of Network Medicine, Harvard Medical School & Brigham and Women’s Hospital, Boston, MA, United States; ^2^ Departments of Environmental Health, Harvard T.H. Chan School of Public Health, Boston, MA, United States; ^3^ Epidemiology, Harvard T.H. Chan School of Public Health, Boston, MA, United States; ^4^ Biostatistics, Harvard T.H. Chan School of Public Health, Boston, MA, United States; ^5^ Vincent Obstetrics and Gynecology, Massachusetts General Hospital, Harvard Medical School, Boston, MA, United States; ^6^ Department of Obstetrics, Gynecology and Reproductive Biology, Harvard Medical School, Boston, MA, United States; ^7^ Nutrition, Harvard T.H. Chan School of Public Health, Boston, MA, United States

**Keywords:** live birth, gestational age, birthweight, perceived stress, *in vitro* fertilization

## Abstract

**Background:**

The epidemiologic literature on women's perceived stress in relation to perinatal outcomes has been inconclusive and does not consider the preconception window of exposure.

**Objective:**

To evaluate whether women's preconception perceived stress is related to live birth, gestational age, and birthweight in a cohort receiving fertility treatment.

**Methods:**

This observational study included women seeking fertility care at the Massachusetts General Hospital (2004–2019). During preconception, women provided information on their psychological stress using the short version of the validated Perceived Stress Scale 4 (PSS-4). We used regression models to evaluate the associations of stress with live birth (*N* = 768 attempting to conceive) and perinatal outcomes (*N* = 413 live births) while adjusting for confounders. Stratified analyses by mode of conception [natural, intrauterine insemination (IUI), and IVF (*in vitro* fertilization)] and selected socioeconomic factors (race, education, and income) were also conducted.

**Results:**

Higher psychological stress was negatively associated with the overall probability of live birth (adjusted RR = 0.95, 95% CI: 0.92, 0.98), particularly among women conceiving using IVF. However, we found no association between women's psychological stress and gestational age and birth weight in the overall analyses and also stratified by mode of conception. Similarly, we observed no differences in women's psychological stress with any of the measured outcomes by socioeconomic factors.

**Discussion:**

These results highlight the importance of considering the preconception window and mode of conception when evaluating the relationship between women's preconception stress and live birth.

## Introduction

The inability to achieve a pregnancy after more than a year of unprotected sex, defined as infertility, has increased during the past decades (1) and it is affecting up to 15% of all couples worldwide (2, 3). Each year, only around 10% of US reproductive aged women try actively to get pregnant (4, 5). Therefore, birth rates have declined in the US general population (6). Moreover, the number of babies born using medically assisted reproduction in the USA is growing and it is estimated to be >250,000 births per year (7–9) and over 1 million over the next 10 years. This increase in assisted reproductive technology (ART) treatments is primarily explained by the delay in women's childbearing in Western Countries (10), given women's participation in the labour force, as well as the options available for using contraceptive methods (11). The Centers for Disease Control and Prevention (CDC)'s 2020 Fertility Clinic Success Rates Report has calculated a 37% live birth rate success following assisted reproductive technologies (12). Higher stress and psychiatric disorders have been found among women who have difficulties conceiving, and those who take medications to treat infertility have reported higher rates of psychological stress as well as anxiety, depression, and overall poor quality of life (13). In addition, experiencing infertility and undergoing infertility treatments, frequently hidden even to family members and friends, can be a source of physical and emotional stress for the couples involved (14, 15).

Among pregnant women, maternal psychological stress has been associated with pregnancy complications, particularly pregnancy loss (16, 17). Some studies have demonstrated a link between maternal stress and preterm birth (18–22). However, the epidemiologic literature on the relationship between maternal stress and birthweight showed mixed results (20, 23, 24). In addition, it is unclear whether the relationships of maternal perceived stress with pregnancy and perinatal outcomes differ by mode of conception. This is particularly important as it could help physicians when choosing the type of infertility treatment at the clinic. For example, intrauterine insemination (IUI) treatment has shown less effectiveness as an infertility treatment compared with *in vitro* fertilization (IVF), so couples undergoing IUI may experience more stress than those undergoing IVF. Perceived stress was positively related to female factor infertility among 286 women and 236 men seeking to become parents through fertility treatment in Canada (25) and female factor infertility is more prevalent in IUI cycles because IVF was traditionally the preferred treatment for couples with male factor infertility (26). However, in a smaller study including 120 Indian couples attending a fertility center, no differences in stress were observed among those undergoing IUI compared with those opting for IVF (27). Also, IUI has been the preferred treatment for unexplained infertility, which can be very stressful not only for the women but for the couple (26). So, additional studies clarifying these associations are needed.

Stress has also been related to important socioeconomic factors often affecting health (28, 29). For example, income and education were more strongly associated with stress in Black adults compared with White adults (30). Also, adverse birth outcomes have been more prevalent among women of color (e.g., Black, Hispanic) (31) and lower income (32). Thus, evaluating perceived stress and birth outcomes by socioeconomic status is also warranted. To address these important knowledge gaps, we aimed to investigate whether self-reported women's preconception psychological stress was associated with birth outcomes among women attending a fertility center. Taking advantage of evaluating this selected group of women seeking fertility care and at high risk of experiencing stress, we also explored whether the association between maternal stress and the examined outcomes differed by mode of conception (natural, IUI, and IVF) and selected the socioeconomic factors (race, education, income). Women in our study were attending a fertility center seeking fertility care as they were unable to conceive after several months/years of trying. This makes our study population at high risk for psychological stress given their concerns of fertility potential. These fertility problems may be a consequence of female or male factors as well as unexplained ones, which can be related to other underlying diseases (all contributing to stress) (26).

## Subjects and methods

### Study population

This study includes women who participated in the Environment and Reproductive Health (EARTH) Study, a prospective cohort gathered with the aim to evaluate environmental and dietary determinants of fertility at the Massachusetts General Hospital (MGH) Fertility Center (33). Between 2004 and 2019, 1,324 women between the ages of 18 and 45 years seeking fertility care at the center were eligible to participate and 991 of those contacted by the research staff enrolled prior to conception in the study (recruitment is not ongoing as the study has already ended). This analysis includes 768 women who self-reported preconception perceived stress at study entry and were followed up. Of these 768 women, 413 had a singleton live birth and had information on perinatal outcomes including gestational age and birthweight (Supplementary Figure S1). The remaining women (*N* = 355) did not have a live birth and thus have no information on perinatal outcomes. Median [interquartile range (IQR)] elapsed time between assessment of stress and perinatal outcome assessment was 352 (263, 494) days.

### Procedures

Data on self-reported preconception perceived stress was collected in a questionnaire at study entry, when women were already assigned an infertility diagnosis. The 768 women included in this analysis underwent one or more medical assisted treatments (*N* = 923 for IUI and *N* = 878 for IVF) or got pregnant naturally (*N* = 163). Of these, 413 had a live birth when participating in the study and have information on perinatal outcomes (the rest of the women did not have a child). A total of 355 women did not have a live birth and thus were not included in the birthweight/gestational age analyses.

### Ethical approval

The Human Subject Committees of the Harvard T.H. Chan School of Public Health and MGH (#1999P008167) approved this study. Trained research study staff collected all the signed informed consents from the study participants.

### Self-reported perceived assessment

We used the short form of the Perceived Stress Scale (PSS-4) to assess perceived stress (34). The women responded concerning the past 3 months: “how often have you felt that you were unable to control the important things in your life?,” “how often have you felt confident about your ability to handle your personal problems?,” “how often have you felt that things were going your way?,” and “how often have you felt difficulties were piling up so high that you could not overcome them?.” The responses included never (0), almost never (1), sometimes (2), fairly often (3), and very often (4) following a Likert scale. Self-reported perceived stress was evaluated as the total scores of each item with a range from 0 (lowest score/stress) to 16 (highest score/stress). We used the total score as a continuous exposure as well as a categorical variable divided in approximate quartiles (Q1 = 0–2, Q2 = 3–4, Q3 = 5–7, Q4 = 8–15; the lowest quartile was the reference group) based on the overall distribution among these women. We used perceived stress as a continuous variable to increase study power when performing stratification. The validity of the PSS-4 to evaluate psychological stress has been previously confirmed when compared with other validated depression and anxiety instruments among 37,451 European subjects (35) and in other smaller studies (36, 37). Furthermore, PSS-4 (short version) has demonstrated high correlation with PSS-10 (long version) (*r* = 0.91) and similar correlations with the PSS-10 with depressive symptoms (*r* = 0.41 and *r* = 0.46, respectively) among Mexican women (38). Similar to other studies (39), the four-item PSS demonstrated acceptable internal consistency (Cronbach's alpha coefficient = .81) among the women in this study.

### Pregnancy and perinatal outcome assessment

Live birth was defined as the birth of a neonate on or after 24 weeks of gestation. The probability of pregnancy and other intermediate reproductive outcomes such as implantation rate were not considered for analyses, as we would not be able to investigate the associations with perceived stress among women conceiving naturally. Gestational age (weeks) was abstracted by trained study staff from delivery records and validated using the American College of Obstetricians and Gynecologists (ACOG) guidelines for dating births following medically assisted reproduction (40). Birthweight (g) was also abstracted from delivery records. Perinatal outcomes were assessed continuously to increase the study power in all the analyses including the stratifications by mode of conception and socioeconomic status.

### Covariate assessment

At study entry, trained study staff collected data on the women's date of birth, weight, and height. We calculated body mass index (BMI) as weight (in kilograms) divided by height (in meters) squared. At enrollment, women completed questionnaires including for information on sociodemographic factors, lifestyle, and medical history. They also completed a comprehensive questionnaire on family, medical, reproductive, and psychological stress, consumer products use, smoking history, and physical activity. Total physical activity was calculated as the sum of vigorous, moderate, and light self-reported leisure exercise (41). Census tract level median family income in the past 12 months (in 2011 inflation-adjusted dollars) from the American Community Survey 2007–2010 was used as a proxy for socioeconomic status. Infertility was diagnosed using the Society of Assisted Reproductive Technology definitions (42). We abstracted mode of conception [natural, IUI, and IVF including intracytoplasmic sperm injection (ICSI)] from medical records and infant sex information was obtained at birth.

### Statistical analysis

We presented the women's and children's demographic as well as reproductive and perinatal characteristics using median ±IQRs or percentages. We also presented the full distribution of the specific PSS-4 items and total score among the examined women using means (SD) and percentiles. We used adjusted log-binomial models with random intercepts to account for correlation between outcomes/cycles among the same woman, to estimate the association between self-reported psychological stress and probabilities of live birth; the results were presented as risk ratios (RR) (95% CI). We used adjusted linear regression models to evaluate the relationships between women's stress and both gestational age and birthweight, and presented the results as betas (95% CI). To allow for better interpretation of the results when using stress as a categorical variable, we presented population marginal means (43). The variables related with both stress and birth endpoints that were not in the causal pathway were considered as confounders (44, 45). Adjusted models included age (years), smoking status (current and ever/never smoked), physical activity (h/week), race (White and Black/Asian/other), education (college degree and other), BMI (kg/m^2^), infertility diagnosis (female factor and male/unexplained), mode of conception (natural, IUI, and IVF including ICSI), and infant sex at birth (female and male, only for perinatal outcomes). We also conducted stratified analyses by mode of conception (natural, IUI, and IVF) and selected socioeconomic indicators including race (White and Black/Asian/other), education (college degree and other), and census-tract median annual household income (<100,000 and ≥100,000$). We used SAS to conduct all the analyses (version 9.4; SAS Institute Inc., Cary, NC, USA).

## Results

The women in this analysis had a median (IQR) BMI of 23.3 kg/m^2^ (21.2, 26.4) and age of 35 years (32, 38). Most women were white (83%), reported never smoking (75%), and were generally highly educated (60% with at least a college degree) (Table 1). Of the 1,964 total treatment cycles and natural pregnancies among the 768 women included, 35% (*N* = 682) resulted in a pregnancy and 28% (*N* = 544) resulted in a live birth. A total of 1,801 (92%) cycles were used in the medically assisted technologies. The women in our study underwent a mean (SD) of 2.58 (2.01) treatment cycles. They had a median (IQR) PSS-4 score of 5 (3, 7) ranging from 0 to 15. A total of 45 women (6%) scored above 10 for PSS-4 and only 4% (*N* = 29) of the women reported a total PSS-4 score of 0. Median (IQR), gestational age (weeks), and birthweight (g) were 39 (38, 40) and 3,317 (3,015, 3,680), respectively (Table 1).

**Table 1:** Women and children’s characteristics in the Environment and Reproductive Health (EARTH) study.

	Women’s characteristics
	(*N* = 768 women, 1,964 cycles) median (IQR or *N* (%)
Age, years	35.0 (32.0, 38.0)
Race, *N* (%)	
White	642 (83)
Black	29 (4)
Asian	68 (9)
Other	29 (4)
Body mass index (BMI), kg/m^2^	23.3 (21.2, 26.4)
Smoking, *N* (%)	
Never smoked	578 (75)
Past smoker	175 (23)
Current smoker	15 (2)
Total physical activity, h/week	5.49 (2.70, 9.70)
Education, *N* (%)	
High school or less	59 (8)
College only	247 (32)
Graduate degree	462 (60)
Census-tract median income^a^, $	100,000 (77,150, 129,000)
Total PSS-4 scores, *n*	5 (3, 7)
Mode of conception^b^, *N* (%)	
Natural	163 (8)
IUI	923 (47)
IVF/ICSI	878 (45)
Live birth^b^, *N* (%)	544 (27)
Initial infertility diagnosis, *N* (%)	
Male factor	189 (25)
Female factor	239 (31)
Unexplained	340 (44)
	Children’s characteristics
	(*N* = 413 singleton pregnancies)
Gender, *N* (%)	
Male	206 (50)
Female	207 (50)
Gestational age, weeks	38 (39, 40)
Birthweight, g	3,317 (3,015, 3,680)

IUI, intrauterine insemination; IVF, *in vitro* fertilization; ICSI, intracytoplasmic sperm injection.

^a^ This variable is based on census tract corresponding to participant address; only available on *N* = 396 maternal participants.

^b^ *N* = 1,964 observations or cycles.

**Figure 1:**
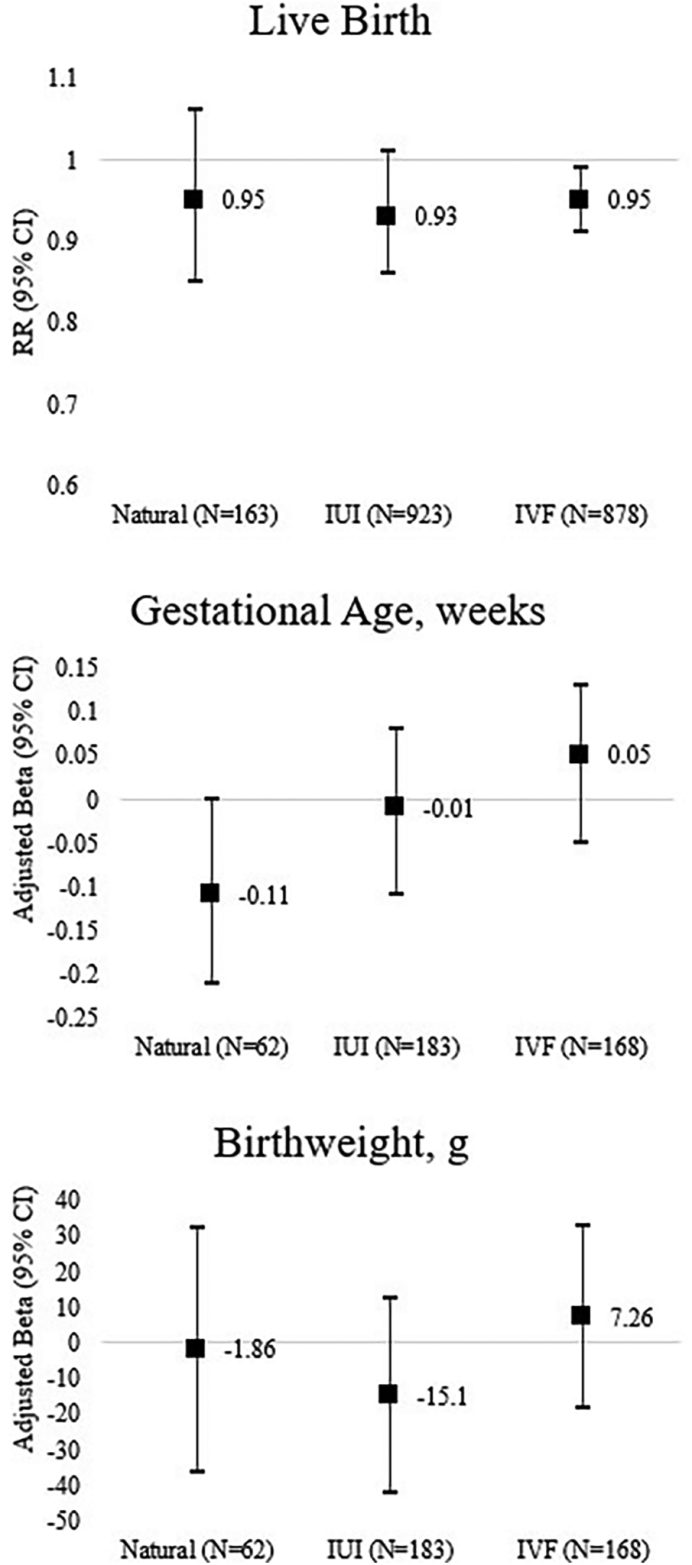
Adjusted maternal and perinatal outcomes by self-reported perceived stress stratified by mode of conception in the Environment and Reproductive Health (EARTH) study. Models are adjusted for age, BMI, race, smoking, education, physical activity, primary infertility diagnosis, and infant sex at birth (only for perinatal outcomes).

We observed a negative association between total PSS-4 scores and live births (Table 2), with monotone decreases over quartiles of stress scores. Specifically, the adjusted marginal means (95% CI) of the probability of having a live birth for women in the first (lowest), second, third, and fourth (highest) quartiles of self-reported perceived stress were 0.41 (0.34, 0.48), 0.38 (0.33, 0.44), 0.34 (0.30, 0.40), and 0.32 (0.26, 0.37), respectively (*p* for trend = 0.02). In the stratified analyses, this association remained among women who conceived using IVF, compared with the naturally or using IUI (Figure 1). We found no association between women's psychological stress and gestational age and birth weight in the overall analyses (Table 2), which was also stratified by mode of conception (Figure 1). Similarly, we observed no differences among the women's psychological stress in association with any of the measured outcomes by socioeconomic factors (Table 3).

**Table 2:** Adjusted^a^ birth outcomes by self-reported perceived stress in the Environment and Reproductive Health (EARTH) study.

	Live birth, proportion	Gestational age, weeks	Birthweight, g
	(*N* = 768 women, 1,964 cycles)	(*N* = 413 singleton pregnancies)	(*N* = 413 singleton pregnancies)
Continuous PSS-4 scores			
	RR (95% CI)	*β* estimate (95% CI)	*β* estimate (95% CI)
	0.95 (0.92, 0.98)	−0.005 (−0.006, 0.05)	−4.24 (−21.3, 12.8)
*p*-Value	0.009	0.85	0.63
Categorical PSS-4 scores	Predicted marginal means (95% CI)		
Q1 (0–2)	0.41 (0.34, 0.48)	38.7 (38.3, 39.1)	3,336 (3,213, 3,459)
Q2 (3–4)	0.38 (0.33, 0.44)	39.3 (38.9, 39.6)	3,391 (3,282, 3,501)
Q3 (5–7)	0.34 (0.30, 0.40)**	38.5 (38.2, 38.9)	3,242 (3,143, 3,342)
Q4 (8–15)	0.32 (0.26, 0.37)*	39.0 (38.7, 39.4)	3,381 (3,267, 3,495)
*p*-trend	0.02	0.85	0.86

^a^ Models are adjusted for age, BMI, race, smoking, education, physical activity, primary infertility diagnosis, mode of conception, and infant sex at birth (only for perinatal outcomes).

* *p*-Value = 0.03 when comparing that quartile with the lowest quartile of exposure.

** *p*-Value = 0.09 when comparing that quartile with the lowest quartile of exposure.

**Table 3:** Adjusted^a^ birth outcomes for continuous self-reported perceived stress score stratified by social determinants in the Environment and Reproductive Health (EARTH) study.

	Race		Education		Income, $	
	White	Black, Asian, other	Undergrad or less	Colleague degree	<100,000	≥100,000
Live birth, proportion	0.97 (0.94, 1.01)	0.97 (0.90, 1.07)	0.96 (0.91, 1.01)	0.98 (0.94, 1.02)	0.95 (0.88, 1.03)	0.94 (0.88, 1.01)
RR (95% CI)						
Gestational age, weeks	0.01 (−0.06, 0.07)	−0.04 (−0.14, 0.06)	−0.01 (−0.10, 0.08)	−0.01 (−0.07, 0.07)	−0.02 (−0.15, 0.10)	−0.01 (−0.09, 0.07)
Beta (95% CI)						
Birthweight, g	−0.16 (−19.5, 19.2)	−24.7 (−59.8, 10.4)	1.51 (−24.9, 28.0)	−8.91 (−31.0, 13.2)	−21.0 (−60.3, 18.6)	−7.95 (−34.6, 18.9)
Beta (95% CI)						

^a^ Models are adjusted for age, BMI, race, smoking, education, physical activity, primary infertility diagnosis, mode of conception, and infant sex at birth (only for perinatal outcomes).

## Discussion

In this observational prospective study, we examined whether women's perceived stress, assessed using the PSS-4 scale during preconception, was associated with live birth and perinatal outcomes in the EARTH study. We also explored this relationship among three groups of women [(1) conceiving naturally; (2) conceiving using IUI; and (3) conceiving using IVF] and also among women belonging to different groups based on socioeconomic factors. We found that psychological stress was negatively associated with the probability of live birth and this association remained for those women conceiving using IVF. We did not find that women's stress was associated with gestational age or birth weight. Similarly, we did not find any associations by socioeconomic status. These results support the association between women's preconception stress and live birth among subfertile women. They also highlight the importance of considering the mode of conception as well as the preconception period when evaluating these relationships. Given the impact of socioeconomic factors on stress as well as the growing number of babies born using ART, future studies to confirm the observed findings in other (and larger) study cohorts are warranted.

Mean total PSS-4 scores among women in this study were similar to those in other reported studies in pregnant women in Spain (mean = 5.43) (35) and France (mean = 5.6) (46). However, participants in China (mean∼6) (47) and Korea (mean∼8) (48) reported higher mean PSS-4 scores. In agreement with our negative associations on women's stress and live birth, peri-implantation and early pregnancy weekly perceived stress (self-reported using the Likert scale) were positively associated with pregnancy loss among 797 US women participating in the Effects of Aspirin in Gestation and Reproduction (EAGeR) trial (16). The same conclusion has been reported in a systematic review and meta-analysis on psychological stress and miscarriage (17). We hypothesize that stress and some pregnancy-related hormones might interact with the peripheral and local immunocompetent cells (certain T-cell subsets, mast cells, or natural killer cells) leading to changes in cytokine production, which can result in an increased risk of miscarriage (49). We did not observe any associations between women's preconception perceived stress and gestational age among women in our study. On the contrary, among the 396 pregnant women from the general population in Ethiopia, perceived stress was associated with pregnancy loss before 12 weeks of gestation (21). Midpregnancy perceived stress was positively associated with preterm birth (gestational age <37 weeks) and low birthweight (<2,500 g) in a group of predominantly Puerto Rican women (*N* = 1,267) from the general population and participating in the research study Proyecto Buena Salud (24). The authors also found that increased stress over the course of pregnancy was positively related to gestational age. In another study, changes in perceived stress scores during pregnancy were correlated with gestational age among 78 women in Texas, with greater decreases in stress scores associated with longer gestational age (22). Stress was also associated with preterm birth in a case-control study including 340 women at Linköping University hospital (18). It has been shown that circulating cortisol, as a biomarker of stress, has been related to preterm birth (50). We also did not observe any association between women's perceived stress and birthweight. Contrary to our results, racial and ethnic disparities in birth outcomes were reported among 93,375 women in Nebraska, with preterm birth and low birthweight being more prevalent among non-Hispanic Black and Hispanic White women, compared with the non-Hispanic white women (51). Women of color are reported to experience double social stress resulting from the interaction between racial and gender discrimination and health and socioeconomic disparity (52). Also, it has been demonstrated that excessive burden was imposed by physiological impacts of stress caused by health disparities associated with chronic stressors, including perceived discrimination, neighborhood stress, daily stress, family stress, acculturative stress, environmental stress, and maternal stress (49). However, we did not find any differences by the examined socioeconomic factors in the relationships of preconception maternal stress with any of the examined birth outcomes.

The association between stress and live birth remained among women who conceived using IVF. One potential explanation is that IVF is a more aggressive infertility treatment compared with IUI (26). However, in a smaller study including 120 Indian couples attending a fertility center, no differences in stress were observed among those undergoing IUI and those opting for IVF (27). An explanation for the null findings in this Indian study is the possibility of lack of power to detect associations as only 60 couples undergoing IUI and 60 undergoing IVF were included. Also, the authors evaluated a different scale for stress, which included questions related to sexual and relationship concerns, among others. We did not observe any associations between preconception stress and birthweight in the main analyses and also for those stratified by mode of conception. Women with higher psychological stress, measured using the Measure of Psychological Stress (MSP-9), during the second trimester (24th–28th weeks) of pregnancy have increased risk for delivering a newborn with macrosomia (birthweight >4,000 g) when compared with women with lower psychological stress in a large cohort of predominantly White women living in an urban area (23). Among 353 pregnant women in Ghana, prenatal maternal stress was associated with reduced birth length, but associations of stress with low birthweight were only observed among girls and not boys (20). Some discrepancies between the results in these studies and our study may be due to different instruments used to collect the information on perceived stress, the window of exposure (preconception vs. prenatal), and the group of women (fertile vs. subfertile). Further evaluation of the relationships between maternal stress and birth outcomes is warranted specially among women belonging to different socioeconomic backgrounds. It is also needed to examine stress before conception given the observed results.

Our study has important limitations. First, extrapolation of these results to women in the general population may be limited given the fact that we enrolled women seeking fertility care. Second, this group of women was mostly White and with high socioeconomic status, which limits out ability to investigate the associations among individuals of color separately, with very low income, etc. Third, misclassification of the exposure by self-reporting perceived stress is possible. Fourth, residual confounding by stress during pregnancy is a concern as we have only one measure of stress during preconception. Fifth, some of the stratified analyses are underpowered given the small sample sizes. Thus, future studies in larger cohorts should be conducted to confirm these results. The strengths include the use of the PSS-4, which is a validated instrument and it has been used worldwide to evaluate psychological stress, as well as the unique opportunity to evaluate stratification by mode of conception. Other strengths of our study include the assessment of both live birth and perinatal outcomes among the same study participants and adjustment of important covariates to reduce the concern of confounding. However, unmeasured confounding (e.g., partner living together, family/social support network, emotional overload, quality of sleep, and general health) is possible as this is an observational study. Related to this, there may be other factors underlying the stress (e.g., depression, trauma, significant life events) that we did not account for.

In conclusion, we found that women's preconception psychological stress was negatively associated with the probability of live birth and this association remained among women using IVF. Women's preconception stress was not, however, associated with gestational or birthweight in the overall or stratified analyses. These results support the association between women's preconception stress and live birth among subfertile women. They also highlight the importance of considering the mode of conception and the preconception period when evaluating these relationships. Given the impact of socioeconomic factors on stress as well as the growing number of babies born using ART, future studies to confirm the observed findings in other (and larger) study cohorts are warranted.

## Data Availability

The datasets presented in this article are not readily available due to privacy and confidentiality reasons. Requests to access the datasets should be directed to lminguez@hsph.harvard.edu.

## Supplementary material

Supplementary Figure S1 Flowchart of the study.
