# Supplementary material for: Women's preconception psychological stress and birth outcomes in a fertility clinic: the EARTH study
Source: Front Glob Womens Health. 2024 Feb 5;5:1293255. doi: 10.3389/fgwh.2024.1293255 (PMC10877713; doi:10.3389/fgwh.2024.1293255)
Supplement: Supplementary Figure S1 — Flowchart of the study. [file Image1.pdf]

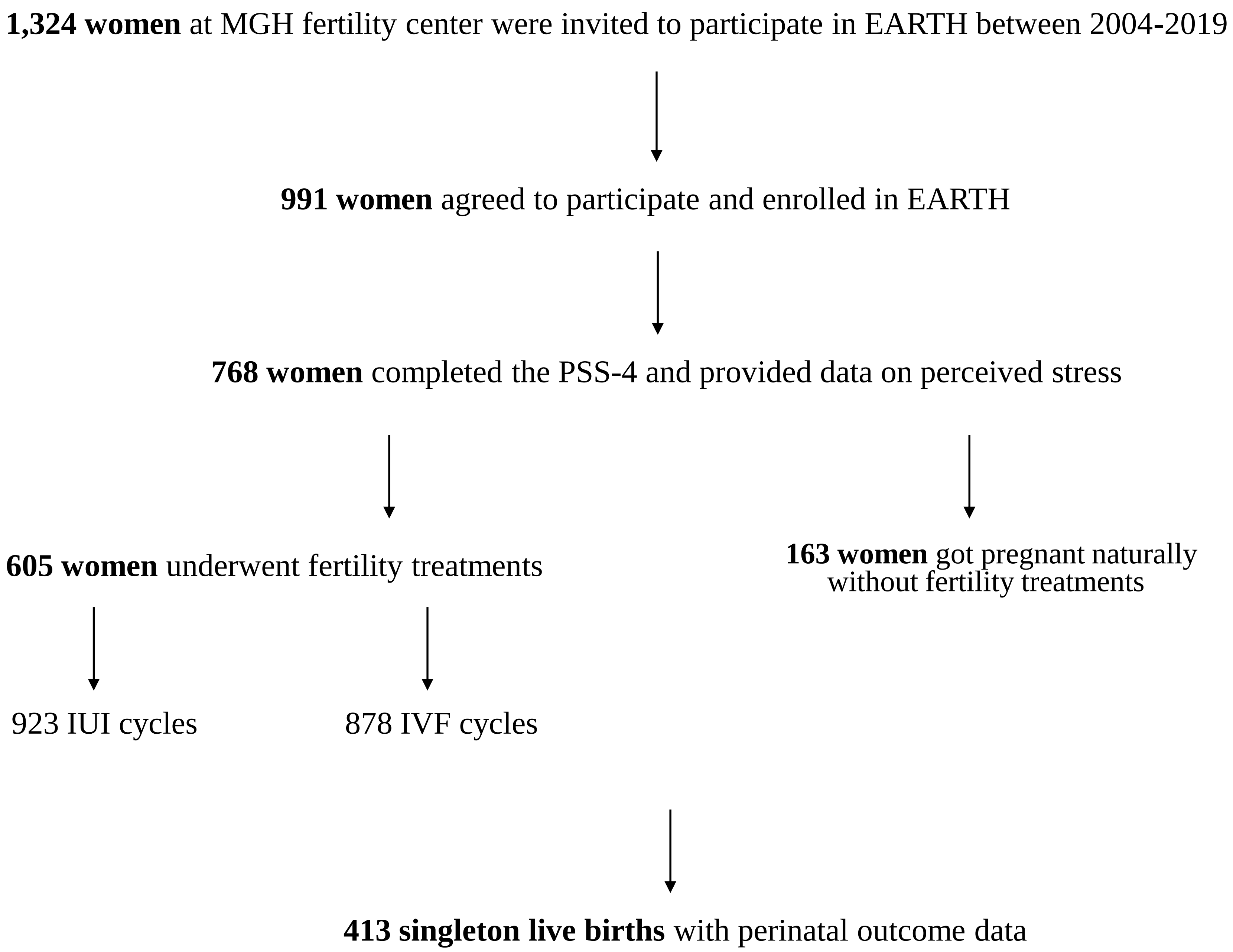
1,324 women at MGH fertility center were invited to participate in EARTH between 2004-2019
991 women agreed to participate and enrolled in EARTH
768 women completed the PSS-4 and provided data on perceived stress
605 women underwent fertility treatments
163 women got pregnant naturally without fertility treatments
923 IUI cycles
878 IVF cycles
413 singleton live births with perinatal outcome data
